# Novel multiplex PCR approach for the detection of *Erysipelothrix rhusiopathiae*, *Streptococcus suis*, and *Staphylococcus hyicus* in swine

**DOI:** 10.3389/fvets.2025.1605316

**Published:** 2025-06-27

**Authors:** A. Arun Prince Milton, Sabia Khan, Kandhan Srinivas, Torik Basar, Zakir Hussain, G. Bhuvana Priya, M. Saminathan, L. H. Puii, Sandeep Ghatak, Samir Das

**Affiliations:** ^1^Division of Animal and Fisheries Sciences, ICAR Research Complex for NEH Region, Umiam, India; ^2^College of Agriculture, Central Agricultural University (Imphal), Kyrdemkulai, India; ^3^ICAR Indian Veterinary Research Institute, Bareilly, India

**Keywords:** multiplex PCR, *Erysipelothrix rhusiopathiae*, *Streptococcus suis*, *Staphylococcus hyicus*, pigs

## Abstract

*Erysipelothrix rhusiopathiae*, *Streptococcus suis*, and *Staphylococcus hyicus* are neglected, emerging and zoonotic pathogens in pigs that share common clinical signs and lesions, particularly septicaemia and skin lesions, highlighting the need for differential diagnosis. Currently, no single diagnostic assay is available to test samples for confirmation of the presence of all three pathogens. To address this, a novel multiplex PCR-based detection assay was optimized and evaluated for the simultaneous detection of *E. rhusiopathiae*, *S. suis*, and *S. hyicus* targeting the, 23S rRNA, *recN*, and *sodA* genes, respectively. The optimized multiplex PCR demonstrated 100% analytical specificity, with a detection limit of 10^4^ copies/μL for *E. rhusiopathiae* and *S. hyicus*, and 10^6^ copies/μL for *S. suis*. The assay was further validated by comparison with previously established uniplex PCR assays and tested on field-collected pig blood samples to assess real-world applicability. The developed assay is rapid, specific and cost-effective and can serve as a handy diagnostic tool for testing suspect samples for these neglected bacterial pathogens of pigs.

## 1 Introduction

Worldwide, the demand for animal-sourced protein foods is consistently increasing, with pork accounting for approximately 35% of global meat production (1). This rising demand has led to the intensification of pig rearing, where higher densities of pigs, combined with stress, poor nutrition and suboptimal husbandry practices, create conditions conducive to the rapid transmission of pathogens among pig herds (2, 3). Infectious diseases in pigs result in significant economic losses due to decreased production, mortality, trade restrictions and food insecurity (1). In addition to being primary constraint in pig production, many of the endemic and emerging infectious diseases of pigs are zoonotic, thereby posing an added burden on human health and underscoring the need for the adoption of a One Health approach (4). In regions like Northeastern India, pig rearing is an integral part of the sociocultural life of the native tribal population, playing a significant role in livelihood upliftment and nutritional security (5). For small holder farmers, who typically maintain 2 to 3 pigs, mortality due to diseases is a primary constraint and can have a substantial impact on their standard of living.

*Erysipelothrix rhusiopathiae*, *Streptococcus suis*, and *Staphylococcus hyicus*, which cause swine erysipelas, streptococcosis, and greasy pig disease, respectively, are emerging, neglected and zoonotic bacterial pathogens of swine (1, 6–9). Moreover, all of these pathogens cause infection in humans through direct contact with carrier pigs or the consumption of under-processed contaminated pork (6, 7, 9).

*Erysipelothrix rhusiopathiae* infection in pigs occurs in three forms: acute septicaemic, sub-acute, and chronic, leading to sudden death, diamond-shaped skin lesions, and arthritic lesions, respectively (7). The global data on burden of swine erysipelas were not abundant based on the perusal of available literature. Swine erysipelas was identified to be the cause of more than 37 per cent of total carcass condemnation in an Italian slaughter house (10). In India, sporadic reports exist from different parts of the country (11–13).

*Staphylococcus hyicus* causes greasy pig disease or exudative epidermitis, in pigs, characterized by destructive skin lesions that range from localized to generalized (6). It has also been reported to cause suppurative pneumonia and septicaemia in pigs (14). Though comprehensive burden estimates are not available globally, a previous study in the adjoining region had reported an occurrence of 9.91% (32).

*Streptococcus suis* infection in pigs typically causes septicaemia, meningitis, endocarditis, arthritis, abscesses and skin lesions including erythema or discoloration (15). *S. suis* has been associated with disease in pigs globally, with China reporting *S. suis* as the most commonly isolated bacterial pathogen from pigs. According to a meta-analysis-based study the prevalence rates in China ranged from 4.2 to 93.7% with a summary estimate of 40.8% (16). Similarly, a meta-analysis done in India provided a summary estimate of 13% (4). The annual losses incurred by *S. suis* to English pig production systems ranged from 100 thousand to about a million pounds, whereas in German, Dutch and Spanish farm, the organism inflicted a mean cost per pig of 1.30 euros, 0.96 euros and 0.60 euros, respectively at the end of each production cycle (17).

These pathogens share most symptoms and lesions in common, particularly septicaemia and skin lesions, highlighting the need for differential diagnosis. Currently, no single diagnostic assay is available to test samples for the confirmation of all three pathogens. Since nucleic acid-based or molecular tests offer rapid results with high sensitivity and specificity, there is a need to optimize a multiplex molecular test for the simultaneous detection of all these pathogens. Therefore, the current study was designed to optimize and evaluate novel multiplex PCR for simultaneous detection of *E. rhusiopathiae*, *S. suis*, and *S. hyicus* in clinical samples.

## 2 Materials and methods

The ethical clearance for the current study was obtained from Institutional Animal Ethics Committee (File no. V-11011(13)/12/2023-CPCSEA-DADF dated December 5, 2023). Single colonies of reference bacterial strains - *E. rhusiopathiae* (ATCC 35456), *S. suis* (ATCC 43765), and *S. hyicus* (VTCC BAA-60) were revived in 5% sheep blood agar, picked, and grown overnight in tryptic soy broth (HiMedia, Mumbai, India). Genomic DNA was then extracted with the QIAamp DNA kit (Qiagen, Hilden, Germany) following the manufacturer’s instructions. Primers targeting the 23S rRNA, *recN*, and *sodA* genes for *Erysipelothrix rhusiopathiae*, *Streptococcus suis*, and *Staphylococcus hyicus*, respectively, were selected from previously described studies, which have been reported as robust and highly specific (18–20). The primer pairs used were as follows: ER1F (5’-GTTCATCTCTCTAATGCACTAC-3′) and ER1R (5’-TGTTGGACTACTAATCGTTTCG-3′) for *E. rhusiopathiae* (amplicon size: 399 bp); SSrecN-F (5’-CTACAAACAGCTCTCTTCT-3′) and SSrecN-R (5’-ACAACAGCCAATTCATGGCGTGATT-3′) for *S. suis* (amplicon size: 336 bp); and STAH-SodI (5’-GCTTATCGCGAATGTTGACCAAT-3′) and STAH-SodII (5’-TCGTGCTGCTGCTTTATCTGAG-3′) for *S. hyicus* (amplicon size: 205 bp). The primers were custom synthesized in Integrated DNA Technologies, New Delhi, India.

The reactions were initially set up in individual uniplex mode and the run conditions were standardized for the laboratory. To facilitate this, three sets of gradient PCRs were set up for all three uniplex assays in temperature range of 51 to 59°C for identifying a common annealing temperature for all three primers. After attaining a common annealing window for successful amplification of all three targets, the multiplex PCR assay was optimized and performed in a 30 μL reaction volume, encompassing 15 μL 2X Emerald AmpGT PCR Mastermix (Takara Bio, Otsu, Japan), 10 pmol (1 μL each) of three primer sets, 1 μL of DNA template (20 ng/μL DNA) and 8 μL of nuclease free water (Thermo Fisher Scientific, Waltham, MA, United States). The PCR cycling conditions performed using a Mastercycler (Eppendorf GmbH, Hamburg, Germany) and included an initial DNA denaturation at 95°C for 3 min, followed by 40 cycles of 94°C for 35 s, 55°C for 40 s, and 72°C for 40 s. After amplification, a final extension step was carried out at 72°C for 5 min. The amplified products were then electrophoresed on a 1.5% agarose gel (Thermo Fisher Scientific), stained with ethidium bromide (Sigma-Aldrich, St. Louis, MO, United States) and run in Tris-Acetate-EDTA buffer (Thermo Fisher Scientific) under a voltage of 90 V for 45 min. The gel was photographed using a documentation system (Vilber Lourmat, Marne-la-Vallée, France).

The analytical specificity of the multiplex PCR was determined using a range of target and non-target reference strains, including: *Erysipelothrix rhusiopathiae* ATCC 35456, *Streptococcus suis* ATCC BAA-853, *Staphylococcus hyicus* VTCC BAA-60, *Staphylococcus aureus* ATCC 25923, *Staphylococcus xylosus* ATCC 29971, *Staphylococcus sciuri* ATCC 29061, *Streptococcus equisimilis* ATCC 12388, *Streptococcus agalactiae* ATCC 13813, *Streptococcus bovis* ATCC 33317, *Campylobacter coli* ATCC 33559, *Brucella abortus* VTCC BAA 465, *Clostridium perfringens* ATCC 13124, *Escherichia coli* ATCC 25922, *Salmonella* Choleraesuis ATCC 10708, and *Yersinia enterocolitica* ATCC 23715. The analytical specificity analysis of the proposed multiplex PCR was repeated at least three times for all of the above strains. The above-mentioned control panel of organisms were chosen in order to evaluate the ability of the assay to exclude phylogenetically close organisms as well as bacterial pathogens of economic importance in porcine husbandry.

The targeted DNA amplicons were cloned into the pTZ57R/T cloning vector (Thermo Fisher Scientific), and plasmids were purified using the QIAprep Spin Miniprep Kit (Qiagen). The concentration of each plasmid was measured using a Nanodrop spectrophotometer (Thermo Fisher Scientific) at an absorbance of 260 nm. The copy numbers of each plasmid were adjusted to 20 ng/μL and quantified using the IDT Copy Number Calculator (Integrated DNA Technologies, Coralville, IA, United States). These standards were then tenfold serially diluted for analytical sensitivity or detection limit analysis. The tenfold serially diluted plasmids were used for performing both uniplex and multiplex PCR to compare the assay performance. Additionally, interference test was performed with the multiplex setup using various combinations of different copy numbers of each template.

A total of 170 blood samples were collected from apparently healthy pigs brought for slaughter to a public service slaughter house following aseptic precautions. The animals were restrained without compromising the animal welfare requirements in slaughter premises. Around 5 mL of blood was collected using a sterile syringe and immediate transferred to EDTA-vacutainers and were suitably labeled. The collected volume was gently mixed and transported to lab for further processing. The genomic DNA was extracted using the QIAamp DNA Blood Kit (Qiagen) and were tested using the developed multiplex PCR and uniplex PCR for all three pathogens. The age of the animals ranged from 5 months to 27 months with a median value of 8 months. Majority of the animals were males (76.47%, 130/170). Animals in this region are predominantly reared in a backyard setup.

## 3 Results and discussion

**Figure 1:**
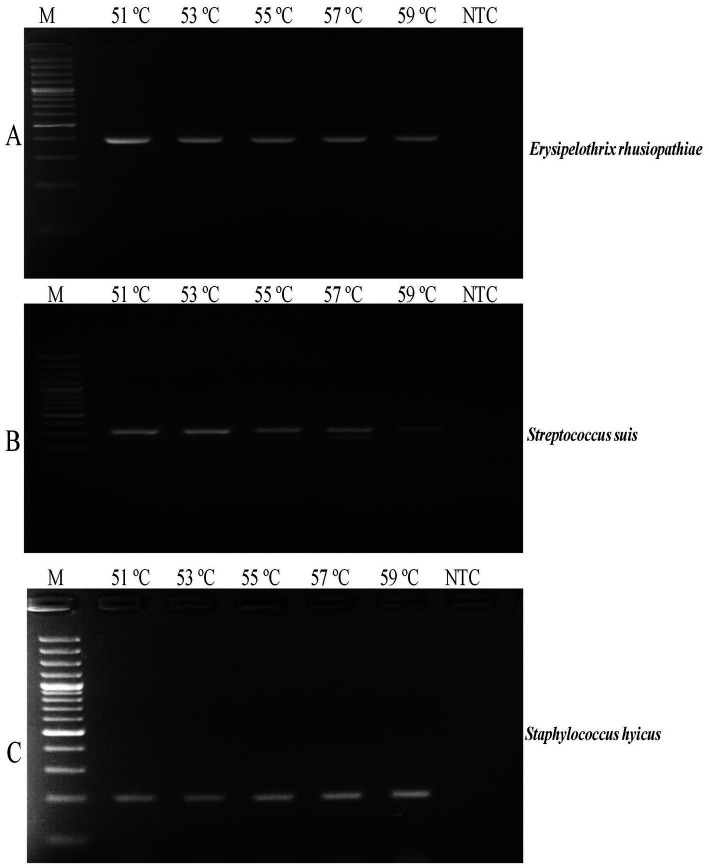
Gradient PCR analysis. **(A)** *E. rhusiopathiae* 399 bp; **(B)** *S. suis* 336 bp; **(C)** *S. hyicus* 205 bp (M: 100 bp DNA Marker (Thermo Fisher Scientific); NTC, No Template Control).

**Figure 2:**
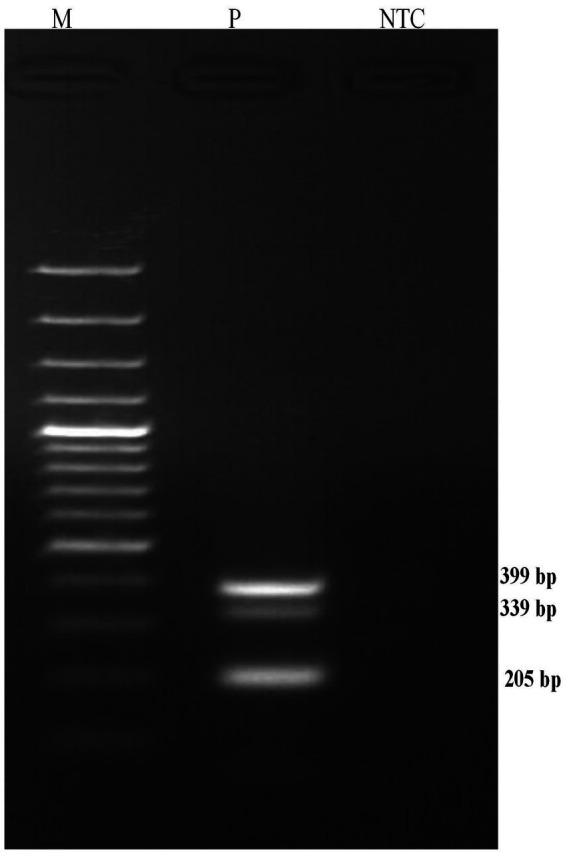
Multiplex PCR assay revealing simultaneous amplification of *E. rhusiopathiae* 399 bp; B. *S. suis* 336 bp; C. *S. hyicus* 205 bp (M: 100 bp DNA Marker (Thermo Fisher Scientific); NTC: No Template Control).

**Figure 3:**
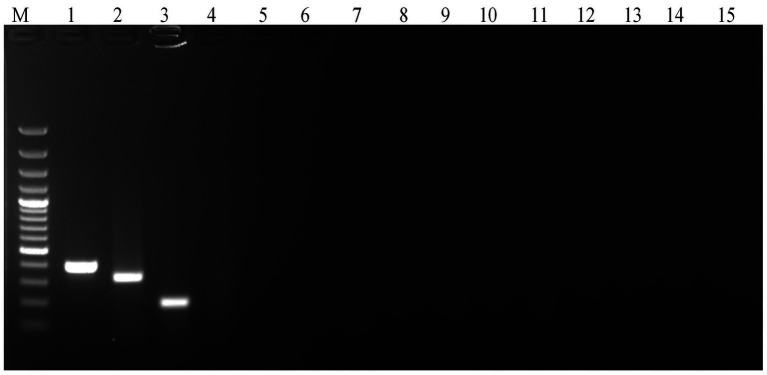
Analytical specificity analysis of multiplex PCR showing amplification only in *E. rhusiopathiae, S. suis* and *S. hyicus* DNA (20 ng) (M: 100 bp DNA Marker (Thermo Fisher Scientific); Lane 1: *Erysipelothrix rhusiopathiae* ATCC 35456, Lane 2: *Streptococcus suis* ATCC BAA-853, Lane 3: *Staphylococcus hyicus* VTCC BAA-60, Lane 4: *Staphylococcus aureus* ATCC 25923, Lane 5: *Staphylococcus xylosus* ATCC 29971, Lane 6: *Staphylococcus sciuri* ATCC 29061, Lane 7: *Streptococcus equisimilis* ATCC 12388, Lane 8: *Streptococcus agalactiae* ATCC 13813, Lane 9: *Streptococcus bovis* ATCC 33317, Lane 10: *Campylobacter coli* ATCC 33559, Lane 11: *Brucella abortus* VTCC BAA 465, Lane 12: *Clostridium perfringens* ATCC 13124, Lane 13: *Escherichia coli* ATCC 25922, Lane 14: *Salmonella* Choleraesuis ATCC 10708, and Lane 15: *Yersinia enterocolitica* ATCC 23715).

**Figure 4:**
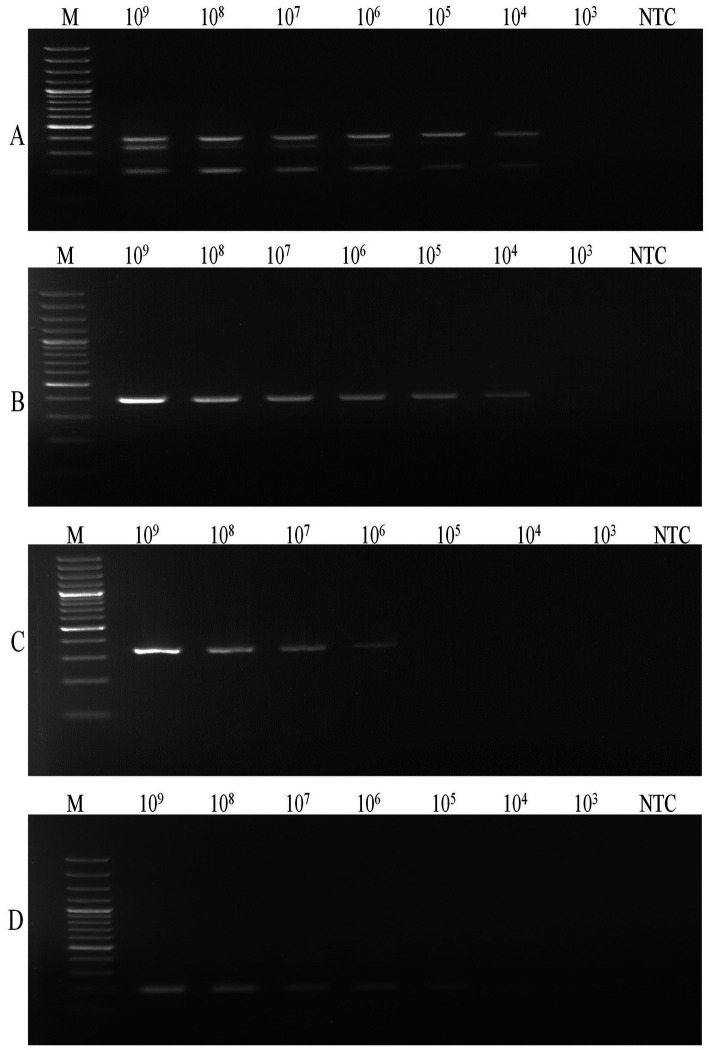
Analytical sensitivity. **(A)** Multiplex PCR; **(B)** Uniplex PCR of *E. rhusiopathiae*; **(C)** Uniplex PCR of *S. suis*; **(D)** Uniplex PCR of *S. hyicus* (M: 100 bp DNA Marker (Thermo Fisher Scientific); Lane 1: 10^9^ copies/μL; Lane 2: 10^9^ copies/μL; Lane 3: 10^9^ copies/μL; Lane 4: 10^9^ copies/μL; Lane 5: 10^9^ copies/μL; Lane 6: 10^9^ copies/μL; Lane 7: 10^9^ copies/μL; NTC: No Template Control).

A common annealing temperature of 55°C for all three primer sets was determined through individual gradient PCR experiments (Figure 1). The multiplex PCR for the concurrent detection of *E. rhusiopathiae*, *S. suis*, and *S. hyicus* was successfully optimized using the specified reagent composition and PCR conditions. The specific primers amplified 399 bp for *E. rhusiopathiae*, 336 bp for *S. suis*, and 205 bp for *S. hyicus*. Agarose gel electrophoresis using a 100 bp plus DNA marker clearly distinguished these amplicons (Figure 2). The optimized multiplex PCR demonstrated 100% analytical specificity, as amplification was observed exclusively for *E. rhusiopathiae*, *S. suis*, and *S. hyicus.* No amplification was detected for other porcine bacterial pathogens, confirming the high analytical specificity of the developed assay (Figure 3). Sensitivity analysis using ten-fold serially diluted DNA standards with known copy numbers (10^9^ copies/ μL to 10^3^ copies/μL) revealed the lowest detectable copy number was 10^4^ copies/μL for *E. rhusiopathiae* and *S. hyicus*, while for *S. suis*, the detection limit was 10^6^ copies/μL (Figure 4). The same detection limits were observed using uniplex PCR for all three pathogens (Figure 4). Additionally, interference test did not detect any event of cross-reactivity or competitive inhibition among primers. Further, the intra-laboratory reproducibility of the developed assay was found to be 100%. Of the 170 field samples tested, 3.52% (6/170) were positive for *S. hyicus* without any infections or coinfections with *E. rhusiopathiae* and *S. suis.* The positive results were identified in six samples obtained from only male animals with age-group ranging from 6 to 9 months.

The Multiplex PCR developed for the detection of *E. rhusiopathiae*, *S. suis*, and *S. hyicus* is a new addition to the existing nucleic acid-based assays available for detecting these pathogens. Nowadays, most animal disease investigation laboratories in developing countries are equipped with PCR systems, including centrifuges for genomic extraction, thermal cyclers for amplification, and gel electrophoresis and documentation systems for result interpretation. Additionally, the use of multiplex PCR for detecting multiple pathogens has become an invaluable tool offering provide rapid and cost-effective results during outbreak investigations. Moreover, these multiplex systems provide enhanced resolution for molecular epidemiological surveillance. Use of established robust primers in multiplex PCR ensures reliability, saves time on primer designing and specificity analysis for a diverse range strains, and enhances efficiency in amplifying multiple pathogens simultaneously. Additionally, for laboratories with economic constraints, limited availability of thermocyclers and reagents could serve as a bottleneck. Diagnostic testing of three different diseases in series could elapse a considerable amount of time where rapid and reliable diagnosis is of paramount importance. Multiplexing considerably reduces the consumption of reagents, power, labour and need for equipment.

A LAMP method for the specific detection of *E. rhusiopathiae* along with few multiplex endpoint and real-time PCR tests for differentiating *E. rhusiopathiae* and *E. tonsillarum* are available in the public domain (21–23). Recently, another multiplex qRT-PCR developed for detecting African swine fever virus, classical swine fever virus, and *E. rhusiopathiae*, with a detection limit of 2.89 × 10^2^ copies/μL for *E. rhusiopathiae* (24). In our study, the detection limit for *E. rhusiopathiae* using end-point PCR was 10^4^ copies/μL without the need for a sophisticated real-time PCR system, while also providing the advantage of multiplexing with other two related pathogens. The primer set (ER1F/ ER1R) targeting 23SrRNA gene of *E. rhusiopathiae* was adapted from Takeshi et al. (19), ensuring accurate amplification of *E. rhusiopathiae* exclusively. Similarly, for *S. hyicus*, several multiplex PCR assays have been developed, particularly for distinguishing different *Staphylococcus* species (25, 26). In a previously developed multiplex PCR developed for the detection of *S. aureus, S. intermedius* and *S. hyicus* in artificially spiked milk, a detection limit of 10^3^ CFU/mL was reported (27). However, these assays were hindered by cross reactivity and the limited number of strains analyzed. To address these issues, Voytenko et al. (20) established a robust PCR targeting the *sodA* gene, which encodes bacterial superoxide dismutase A. In our multiplex PCR, we have adapted their primer set (STAH-SodI/ STAH-SodII). The *sodA* gene, exhibiting minimal sequence variation across strains isolated from different countries and time periods, was demonstrated to be a reliable target for the specific molecular detection of *S. hyicus* (20). *S. suis* has been detected using various PCR, multiplex PCR and LAMP assays (28–30). Although the PCR assays developed by Okwumabua et al. (30), targeting the *gdh* gene encoding glutamate dehydrogenase, and by Marois et al. (29), targeting the 16S rRNA gene, have been widely applied in clinical settings, originally intended to identify all 35 serotypes, without excluding those that have been removed after reclassification. In the present study, we utilized a novel primer set (SSrecN-F/ SSrecN-R) targeting the *recN* gene (encoding recombination or repair protein) of *S. suis*, as used in the PCR method developed by Ishida et al. (18). This primer sets which corresponds to the current reclassification of *S. suis*. This primer set aligns with the updated reclassification of *S. suis*, offering greater accuracy and reliability. A recently developed LAMP based assay targeting *thrA* gene demonstrated a detection limit of 10^3^–10^5^ CFU/swab in brain and joint tissues (28). Notably, our study showed a detection limit of 10^6^ copies/μL of *S. suis*, despite using endpoint multiplex PCR. The difference in detection limit can be attributed to different amplification dynamics of the primers influenced by primer design and nucleic acid extraction procedures. Interestingly, the multiplex assay developed in this study maintained the same LoD values identified in uniplex setups indicating that the assay had limited influence of either PCR drift (stochastic fluctuations in availability of reagents) or PCR selection (difference in the physico-chemical dynamics of the target sequences) which are usually experienced while upgrading from a uniplex to a multiplex setup (31).

The findings and inferences drawn from the preliminary study are subject to due consideration of the following limitations. Due to limited resources and sparse occurrence of the diseases in the study area, diagnostic sensitivity and diagnostic specificity could not be evaluated. Known references, but not field samples were used to evaluate analytical specificity. The assay also featured variable LoD values for different targets but maintained same LoD values as of uniplex assays. On retrospective perusal of literature, limited availability of validation data related to these three previously reported uniplex assays served as a bottleneck for comparison and subsequent discussion. Moreover, the field samples tested to validate the assay were from apparently healthy animals brought to a public slaughter house, and consequentially yielded limited number of positive results owing to sporadic occurrence of these pathogens in the study area.

## 4 Conclusion

In conclusion, we successfully optimized a multiplex PCR assay for concurrent and differential detection of *E. rhusiopathiae*, *S. suis*, and *S. hyicus* in suspected pig tissue samples. This study is the first to develop such a simplified and integrated PCR approach for diagnosing these neglected bacterial and zoonotic pathogens in pigs. This assay serves as an essential diagnostic method for the early detection of bacterial diseases in pigs, enabling timely antimicrobial treatment to reduce mortality and prevent further spread within and among neighboring herds. Further validation studies involving clinical samples or outbreak settings or high prevalence areas are required to extensively evaluate the newly developed assay for field-level implementation. Additionally, exploring alternative platforms such as point-of-care tests and real-time multiplex assays could help address the needs of both resource-limited and well-equipped diagnostic laboratories.

## Data Availability

The original contributions presented in the study are included in the article/supplementary material, further inquiries can be directed to the corresponding authors.
